# A New Steel Defect Detection Algorithm Based on Deep Learning

**DOI:** 10.1155/2021/5592878

**Published:** 2021-03-17

**Authors:** Weidong Zhao, Feng Chen, Hancheng Huang, Dan Li, Wei Cheng

**Affiliations:** College of Electrical and Information Engineering, Anhui University of Technology, Maanshan 243000, China

## Abstract

In recent years, more and more scholars devoted themselves to the research of the target detection algorithm due to the continuous development of deep learning. Among them, the detection and recognition of small and complex targets are still a problem to be solved. The authors of this article have understood the shortcomings of the deep learning detection algorithm in detecting small and complex defect targets and would like to share a new improved target detection algorithm in steel surface defect detection. The steel surface defects will affect the quality of steel seriously. We find that most of the current detection algorithms for NEU-DET dataset detection accuracy are low, so we choose to verify a steel surface defect detection algorithm based on machine vision on this dataset for the problem of defect detection in steel production. A series of improvement measures are carried out in the traditional Faster R-CNN algorithm, such as reconstructing the network structure of Faster R-CNN. Based on the small features of the target, we train the network with multiscale fusion. For the complex features of the target, we replace part of the conventional convolution network with a deformable convolution network. The experimental results show that the deep learning network model trained by the proposed method has good detection performance, and the mean average precision is 0.752, which is 0.128 higher than the original algorithm. Among them, the average precision of crazing, inclusion, patches, pitted surface, rolled in scale and scratches is 0.501, 0.791, 0.792, 0.874, 0.649, and 0.905, respectively. The detection method is able to identify small target defects on the steel surface effectively, which can provide a reference for the automatic detection of steel defects.

## 1. Introduction

**Figure 1:**
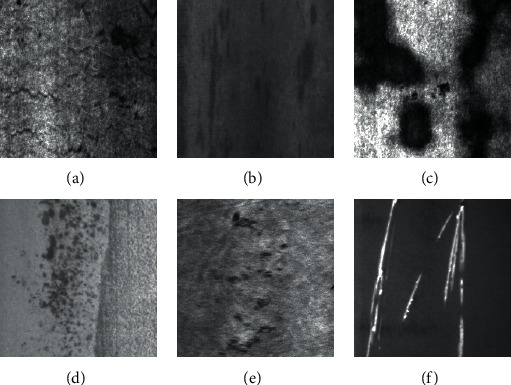
Steel surface defect image. (a) Crazing, (b) inclusions, (c) patches, (d) pitted surface, (e) rolled in scale, and (f) scratches.

It will produce a variety of defects in the steel rolling process due to the environment, production process, and other restrictions, which have a certain influence on the wear resistance and toughness of steel. For example, it will produce crack defects during steel heating due to improper processing or the quality of the steel. It may lead to inclusion defects on the steel surface if some nonmetallic inclusions are rolled into the steel surface or the rolling mill environment is not clean. It may produce plaque defects if the steel surface has corrosion, emulsification, and other conditions. Due to the high temperature and long time, it will lead to surface defects pitted surface, that is, coarse grains and other phenomena. There will be an oxide layer and rust on the surface when the steel is exposed to air for a long time, and then the steel will contact with the air at a high temperature to form the rolling scale [1] in the rolling process. The steel surface may show a scratch phenomenon because the steel oxide scale or other foreign matters contact with the high temperature rolled piece and scratch. The images of several common defects on the steel surface are shown in Figure 1, including crazing, inclusions, patches, and pitted surface, rolled in scale and scratches.

The traditional steel surface defect detection [2–4] is completed by manual visual inspection combined with traditional machine vision [5–7]. There are some shortcomings in manual testing, such as low confidence and high labor intensity. The traditional target detection [8] selects candidate regions on a given image; then, the features are extracted manually and the trained classifier is used for classification. This method has high time complexity and low precision and is difficult to meet the actual production needs of the steel industry. With the continuous development of the convolutional neural network [9], target detection based on deep learning has become the mainstream surface defect detection method. Qu et al. [10] designed an improved Gabor filter optimization method to complete the defect detection of strip steel surface by comparing the shortcomings of the traditional gray projection model and HTM model. Xu et al. [11] proposed a rapid detection method for visual, significant defects based on spectral residual, which realized the accurate detection and classification of various defects on the strip surface. Versaci et al. [12] was committed to the development of ultrasonic nondestructive testing and classification technology based on the continuous wave and proposed a fuzzy similarity based method. The above detection and classification methods for steel surface defects are mainly to extract the shallow features of the target, so the adaptability of this method in the practical application of steel surface defect detection is poor because the defects on the steel surface are complex and uncertain. In contrast, deep learning has multilayer perceptron with multiple hidden layers, which can form more abstract category features by combining low-level features. Therefore, the steel surface defect detection method based on deep learning is widely used in steel production, and more scholars begin to improve and perfect it [13, 14]. Fu et al. [15] proposed a convolutional neural network model which emphasizes the training of the underlying features and realized the rapid and accurate classification of steel surface defects by combining with multiple receptive fields. Lv et al. [16] proposed a new end-to-end defect detection network (EDDN) to deal with different types of steel surface defects. Marco et al. [17] discussed the improved methods of steel surface defect detection and classification by comparing the traditional machine learning model and deep learning model in steel defect classification. Song et al. [18] proposed a new saliency detection method based on an encoder-decoder residual network (EDR-Net). In the coding stage, the full-convolution neural network is used to extract defect features, and an attention mechanism is integrated to accelerate the convergence of the model. As the mainstream target detection framework, Faster R-CNN is used in surface defect detection of steel and metal widely. Wang et al. [19] proposed a Faster R-CNN algorithm integrating multilevel features, which solved the problem of detection of diverse and random defects on the surface of metal plate and strip. Dai et al. [20] designed a defect detection algorithm based on improved Faster R-CNN in order to solve the problems of limitations and low precision of workpiece surface defect detection, which improves the detection performance of defects compared with traditional methods. The research of automatic detection of steel surface defects is focused on in this paper. At the first, the ResNet-50 [21–24] network is reconstructed by deformable convolution as the prefeature extraction network of Faster R-CNN [25–27]. Then, the fixed convolution layer and pooling layer are replaced with deformable convolution layer and deformable pooling layer. Finally, the FPN [28–30] network is used for multifeature fusion, and the soft nonmaximum suppression (soft NMS) [31] is used to reduce the confidence of the detection frame larger than the threshold, so as to alleviate the situation of the target missing detection. According to the test results on the open dataset NEU-DET, the proposed algorithm can effectively detect a variety of defects on steel surface, which is higher than the ordinary steel surface detection algorithms in accuracy.

## 2. Steel Defect Detection Method

### 2.1. Faster R-CNN Model

**Figure 2:**
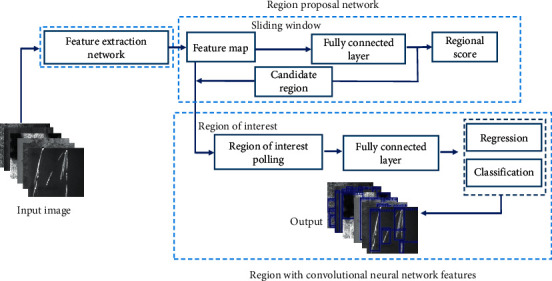
The structure diagram of Faster R-CNN.

Faster R-CNN is the mainstream two-stage target detection algorithm, which is used in face detection and defect detection widely. The detection accuracy of Faster R-CNN is higher than SSD [32, 33], Fast R-CNN [34], and other algorithms verified on multiple datasets. Faster R-CNN algorithm structure includes feature extraction network, region proposal network (RPN), and regional convolution neural network mainly. The algorithm structure is shown in Figure 2. Firstly, the features of the input image are extracted by the feature extraction network; then the extracted features are shared to the RPN network and R-CNN network. Next, the region of interest (ROI) of the image is extracted. Finally, the detection results are output through ROI pooling and fully connected layer.

### 2.2. Steel Defect Detection Algorithm Based on Improved Faster R-CNN

**Figure 3:**
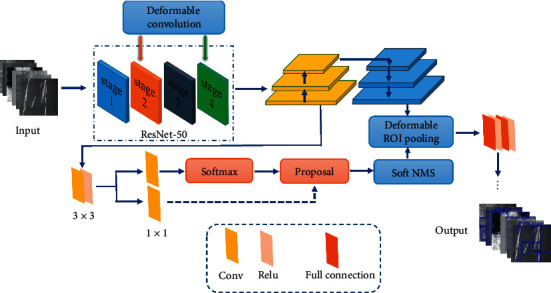
The improved method of Faster R-CNN.

In this paper, a steel defect detection algorithm based on the deformable network [35] and multiscale feature fusion is proposed. Faster R-CNN is used as the basic framework, which is composed of feature extraction network, regional recommendation network, and detection network. The improved method is shown in Figure 3, in which ResNet-50 is used as a feature extraction network. Firstly, the deformable convolution is used to reconstruct the ResNet-50 network. Secondly, the feature pyramid network FPN is used to fuse the multiscale features, and the fixed region of interest (ROI) pooling layer is replaced by the variable pooling layer. Finally, the soft nonmaximum value suppression algorithm is used to suppress the detection frame with obvious overlap with the highest score detection frame.

**Figure 4:**
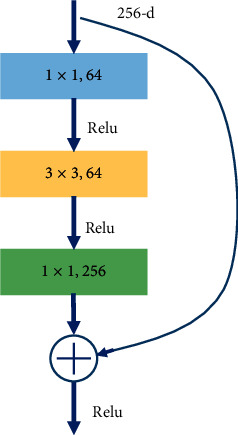
The bottleneck structure of ResNet-50 network.

**Figure 5:**
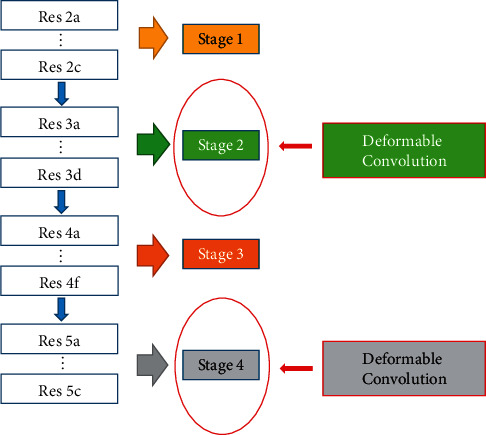
Reconstruction of ResNet-50 using variable convolution.

The conventional convolution network or fully connected network will lead to the loss of partial information in information transmission, which can not train a deep network. However, the ResNet transmits the input information to the output directly, which can protect the integrity of information and reduce the learning difficulty. Therefore, ResNet-50 is selected as the feature extraction network in this paper. A bottleneck module is used in ResNet-50, as shown in Figure 4. It reduces the amount of computation and speeds up the operation by 1 × 1 convolution. Firstly, a 1 × 1 convolution is used to reduce the input dimension characteristic matrix channel from 256 to 64. In the middle, a 3 × 3 convolution is used to deepen the network. Finally, the dimension is restored by the 1 × 1 convolution. Aiming at the small and complex defects on the steel surface, convolution layers of stages 2 and stage 4 (as shown in Figure 5) are reconstructed by deformable convolution to improve the ability to extract features.

### 2.3. Deformable Network

The block convolution kernels of fixed size are often used in the conventional convolution neural network while building model transformation, which is usually limited to fixed geometry structure. The convolution unit is weak in feature extraction of convolution layer with fixed sampling points, which makes the network difficult to adapt to geometric transformation. To solve the above problems, Dai et al. [36] introduced deformable convolution and deformable pooling into convolution neural network to enhance the modeling ability of the network. The end-to-end training is carried out through standard backpropagation to generate a deformable convolution network. There is no fixed geometric structure due to the different shapes of steel surface defects. Therefore, the idea of deformable convolution is introduced to reconstruct ResNet-50 for the poor adaptability to the ability of unknown changes and weak generalization, so as to improve the recognition ability of the neural network for irregular targets.

**Figure 6:**
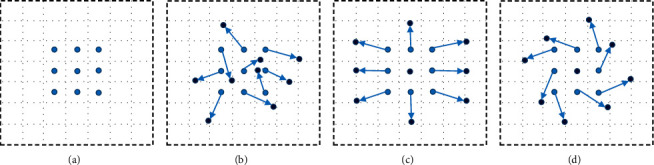
Four sampling methods of convolution.

The offset variable is added to each element of the convolution kernel in deformable convolution, which is calculated by standard convolution unit. Therefore, the range in the training process can be expanded by the convolution kernel. In addition, the size and position can be adjusted dynamically to adapt to the geometric deformation of different objects in accordance with the information of the image recognized. A convolution layer is added to the input feature map extracted by the conventional convolution kernel to obtain the deformation offset of deformable convolution. The convolution kernel is used to generate feature map and offset to realize synchronous learning. The various forms of the conventional convolution kernel and the deformable convolution kernel are shown in Figure 6, where Figure 6(a) represents the conventional convolution kernel sampling point, Figure 6(b) represents the deformable convolution kernel sampling point after adding offset variables, Figures 6(c) and 6(d) are special cases of deformable convolution kernel sampling.

In the conventional convolution kernel, the convolution of each pixel in the input image at position of *p*_0_ is expressed as follows:

(1) $$\begin{array}{c} y\left(p_{0}\right)=\underset{p_{n\in R}}{\sum }w\left(p_{n}\right)x\left(p_{0}+p_{n}\right). \end{array}$$

An offset variable {Δ*p*_*n*_*|n*=1,2,…, *N*} is introduced into the deformable convolution; the deformation convolution of each pixel in the input image is expressed as follows:

(2) $$\begin{array}{c} y\left(p_{0}\right)=\underset{p_{n\in R}}{\sum }w\left(p_{n}\right)x\left(p_{0}+p_{n}+\Delta p_{n}\right). \end{array}$$

**Figure 7:**
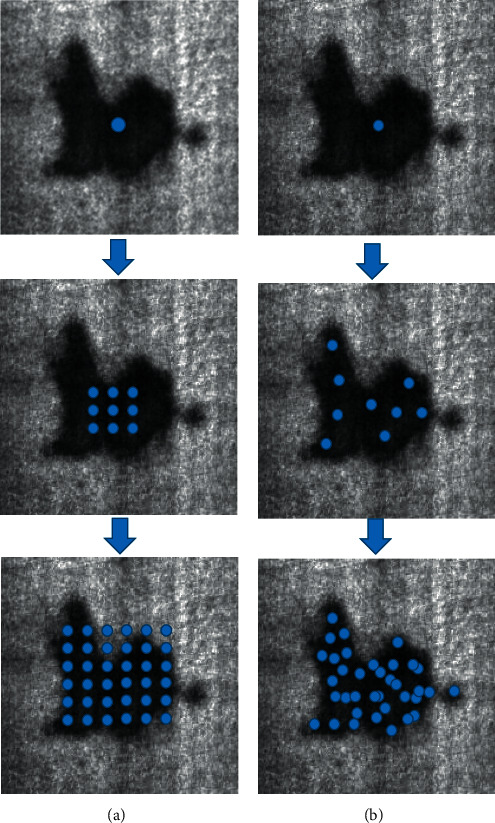
The calculation process of conventional and deformable convolution.

In formulas (1) and (2) [36], *y* is the output feature map, *x* is the input feature map, *p*_0_ is the position of pixel, *w* is the parameter of weight, *p*_*n*_ is any pixel in convolution, and Δ*p*_*n*_ is the offset value. The bias domain of deformable convolution points to the sampling point with a strong purpose and outputs more feature information because the bias matrix makes the sampling position of convolution transform freely, so when the effect of deformable convolution is stacked, the feature extraction ability is greatly improved. Taking a 3 × 3 convolution as an example, the author uses Figure 7 to show the difference between conventional convolution and deformable convolution. The sampling position of the conventional convolution on the target is fixed, as shown in Figure 7(a), while the receptive field can be learned adaptively by the deformable convolution during calculation in Figure 7(b), which has a strong adaptive extraction ability for complex and irregular targets. It can be adjusted adaptively under the shape and size of the target. Therefore, this adaptive learning receptive field is very necessary in steel defect detection.

**Figure 8:**
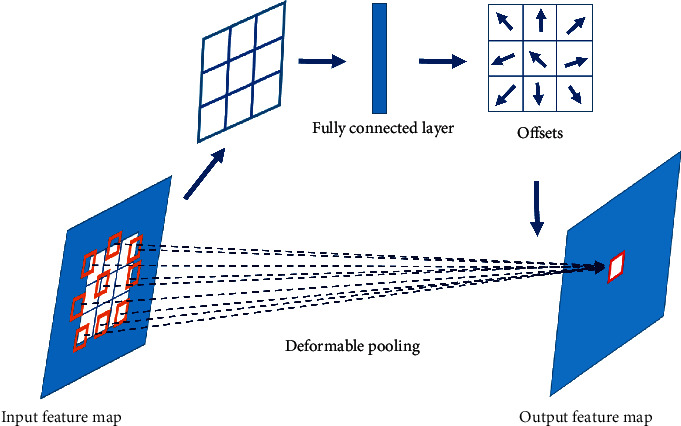
The network structure of deformable pooling.

ROI pooling module is introduced to maximize the pool of the proposed regions after segmentation in order to map the input regions of different sizes into feature vectors of the same length and output a fixed size feature map. The fixed ROI pooling module is replaced with the deformable ROI pooling module in the proposed method in order to enhance the modeling ability of the network for geometric transformation and increase the pool area of defects to obtain the location accurately of complex defects on the steel surface. The network structure of deformable ROI pooling is shown in Figure 8.

The regions of interest are divided into blocks of *k* × *k* by ROI pooling, and the size of each area is *w* × *h*. The output of normal ROI pooling [36] is as follows:

(3) $$\begin{array}{c} y\left(i,j\right)=\underset{p\in \text{bin}\left(i,j\right)}{\sum }\frac{x\left(p_{0}+p\right)}{n_{i,j}}. \end{array}$$

As can be seen from Figure 7, in the deformable ROI pooling, ROI pooling generates convolution feature graphs and then generates regularized offsets with the size of 2 × *k* · *k* at each position through the full-connection layer. Finally, the region with enhanced offset is pooled to generate the output feature map. Referring to the idea of deformable convolution, the expression of deformable ROI pooling [36] is as follows:

(4) $$\begin{array}{c} y\left(i,j\right)=\underset{p\in \text{bin}\left(i,j\right)}{\sum }\frac{x\left(p_{0}+p+\Delta p_{i,j}\right)}{n_{i,j}}, \end{array}$$

where *y*(*i*, *j*) is the output characteristic graph after pooling, *x* is the input feature map, *p*_0_ is the upper left corner pixel of ROI, *p* is a pixel at any position, bin(*i*, *j*) is the set of horizontal and vertical coordinates of the pixel, *n*_*i*,*j*_ is the pixel value of the grid, and Δ*p*_*i*,*j*_ is the offset at each location, where 0 ≤ *i*,  *j* < *k*.

### 2.4. Multiscale Feature Fusion

**Figure 9:**
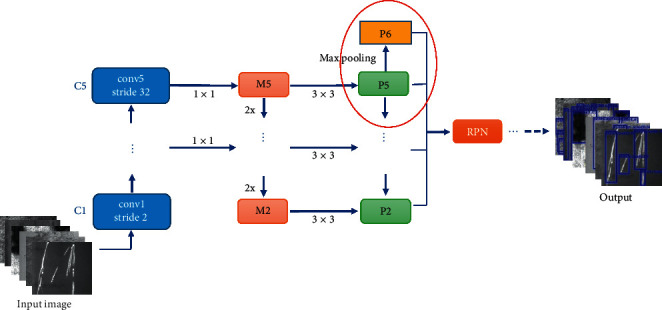
The network structure diagram of multiscale feature fusion.

The features of the last layer are used in RPN because the top-level features of the network have the strongest semantic information in Faster R-CNN, but this idea is not suitable for small target detection. The feature pyramid network FPN is introduced into Faster R-CNN to perform degree scale fusion operation on the feature map in order to improve the ability of the network to detect small area defects on the steel surface. The structure is shown in Figure 9.

The characteristics of all layers are integrated into FPN and the feature activated output of the last residual structure of conv2_x, conv3_x, conv4_x, and conv5_x phases in ResNet-50 network is used as input. The channel number of C2-C5 is reduced to 256 by the 1 × 1 convolution, and M2-M5 are obtained. The output P2-P5 of FPN is obtained by adding the same size of shallow feature map and deep feature map by upsampling, and then the 3 × 3 convolution is used. The P6 is obtained by downsampling with the two largest pools on P5, and the multiscale fusion feature combination is output. The target candidate frame of interest is generated by using the fused features in RPN, and the detection results are obtained by classification.

### 2.5. Soft Nonmaximum Suppression

Nonmaximum suppression (NMS) is used as a postprocessing method commonly to eliminate duplicate frames and reduce the false detection rate in the target detection. However, other similar targets attached to the box with higher prediction score are removed by the NMS, which will lead to missed detection and false detection of similar targets with close proximity and overlapping. The reduction range of the prediction accuracy of dense targets nearby is determined by soft NMS in light of the intersection over union (IOU) [36] of the highest score box, so as to improve the prediction accuracy of dense targets. For example, the IOU of the detection frames *A* and *B* [35] is the following:

(5) $$\begin{array}{c} \text{IOU}=\frac{A\cap^{}B}{A\cup B}=\frac{S_{U}}{S_{A}+S_{B}-S_{U}}, \end{array}$$

where *S*_*A*_, *S*_*B*_ are the areas of the detection frame *A* and *B*, respectively, and *S*_*U*_ is the overlapping area of the detection frames *A* and *B*. The expressions of traditional NMS and Soft NMS algorithms are as follows [35]:

(6) $$\begin{array}{c} S_{i}=\left\{\begin{array}{c} S_{i},\text{ IOU}\left(M,\;b_{i}\right)<\;N_{t},\\ 0,\text{ IOU}\left(M,b_{i}\right)\;\geq \;N_{t}, \end{array}\right) \end{array}$$

(7) $$\begin{array}{c} S_{i}=\left\{\begin{array}{c} S_{i},\text{ IOU}\left(M,b_{i}\right)<\;N_{t},\\ S_{i}\left(1-\text{IOU}\left(M,\;b_{i}\right)\right),\text{ IOU}\left(M,b_{i}\right)\geq \;N_{t}, \end{array}\right) \end{array}$$

where *S*_*i*_ is the score of the detection box *i*, *M* is the maximum score box, *b*_*i*_ is the collection of detection frames *i*, *N*_*t*_ is the threshold set. The NMS sets the score of the detection frame whose IOU is greater than the threshold value to 0, while the soft NMS attenuates the score of the detection frame whose IOU is greater than the threshold value, which can alleviate the problems of missed detection and false detection of targets. The IOU threshold of soft NMS is 0.5 and the minimum score is 0.05.

### 2.6. Experimental Results and Analysis

#### 2.6.1. Experimental Environment and Parameter Setting

The experimental platform is shown in Table 1 and the initialization model parameters of the experiment are shown in Table 2.

The average precision (AP) is used as the evaluation index of each defect detection in this experiment after the model training is completed, and the mean average precision (mAP) is used as the evaluation index of the whole model performance. The definitions of precision (P), AP, and mAP [37] are shown as follows:

(8) $$\begin{array}{c} \text{P}=\frac{\text{TP}}{\text{TP}+\text{FP}}, \end{array}$$

(9) $$\begin{array}{c} \text{AP}=\frac{1}{11}\underset{r\left\{0,\;0.1,\;0.2,\cdots ,1\right\}}{\sum }\rho_{\text{interp}\left(r\right)}, \end{array}$$

(10) $$\begin{array}{c} \text{mAP}=\frac{1}{n_{j}\sum_{j=1}^{n_{j}}{\text{AP}}_{j}}, \end{array}$$

where TP is the number of samples with correct detection, FP is the number of negative samples with detection, and *r* is the value of recall (*R*). The definition of recall rate is shown in formula (10), *ρ*_interp(*r*)_ can be expressed as formula (11) [37], *j* is the category index, *n*_*j*_ is the total number of categories, and AP_*j*_ is the average precision of each category:

(11) $$\begin{array}{c} \text{R}=\frac{\text{TP}}{\text{TP}+\text{FN}}, \end{array}$$

(12) $$\begin{array}{c} \rho_{\text{interp}\left(r\right)}={\;}_{\tilde{r}:\tilde{r}\geq r}^{\max\rho \left(\tilde{r}\right)}, \end{array}$$

where FN is the number of samples with error detection and $\rho \left(\tilde{r}\right)$ is the recall rate of $\tilde{r}$.

The results and discussion may be presented separately, or in one combined section, and may optionally be divided into headed subsections.

### 2.7. Analysis of Experimental Results

**Figure 10:**
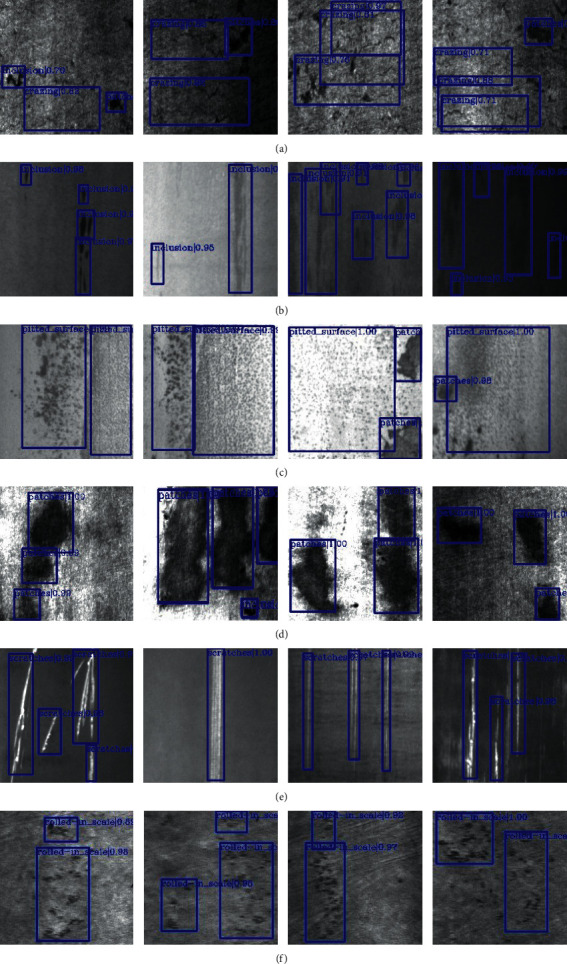
The detection results of the proposed algorithm on all defects. (a) Crazing. (b) Inclusion. (c) Patches. (d) Pitted_surface. (e) Rolled-in_scale. (f) Scratches.

The proposed algorithm is verified on the open dataset of steel defects, and the experimental results are shown in Table 3. The test dataset is NEU-DET, which is the open dataset of Northeast University. There are 1800 pictures in total. Among them, there are 300 pictures of six kinds of defects. The six defects are marked as “crazing,” “inclusion,” “patches,” “pitted_surface,” “rolled-in_scale,” and “scratches.” The test results are shown in Figure 10, and four different pictures are listed for each defect result.

It is not hard to see that the average accuracy of “scratch” is the highest and the “pitted_ surface” is the second in Table 3, which can reach 90.5% and 87.4%, respectively. The average accuracy of “crashing” is the lowest, which is 50.1%, and the overall mean average accuracy is 75.2%. As shown in Figure 10, the defect of “inclusion,” “patches,” “pitted_surface,” “rolled-in_scale,” and “scratches” can be detected and located accurately. The proposed algorithm still has the possibility of optimizing the detection of small targets due to the fuzzy and small size of crazing defects and a small number of missed detection. It can be seen from Figures 10(a)–10(c) that the defect target can also be detected in the case of light or dark, which indicates that the algorithm proposed in this paper is also suitable for defect detection on steel surface under complex background. Generally speaking, the proposed algorithm has high test effectiveness on the NEU-DET dataset.

### 2.8. Ablation Experiments

The proposed scheme is compared with different improved schemes on the same dataset in order to verify the effectiveness of it. The specific improvement schemes are shown in Table 4.

The five specific schemes in Table 4 are as follows:

1. Scheme 1 is the original Faster R-CNN model.
2. Scheme 2 uses ResNet-50 as the feature extraction network of Faster R-CNN.
3. Scheme 3 introduces a deformable network on the basis of ResNet-50 as the feature extraction network of Faster R-CNN.
4. Scheme 4 adds the feature pyramid network on the basis of Scheme 5.
5. Scheme 5 replaces nonmaximum suppression scheme with a soft nonmaximum on the basis of Scheme 4.

**Figure 11:**
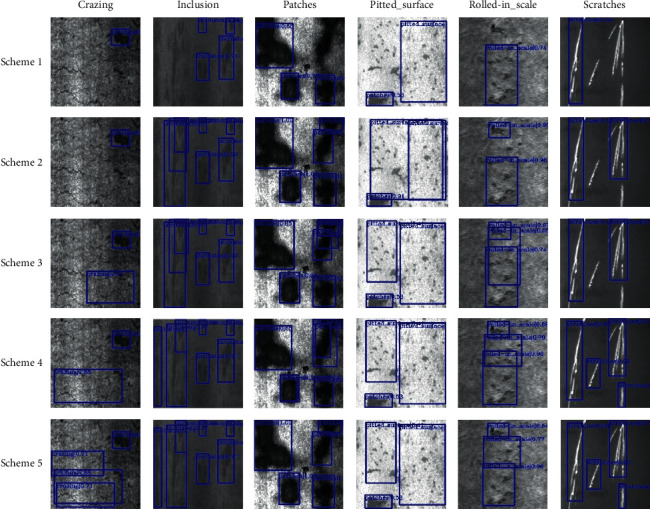
The results of different schemes.

The final test results of the four schemes are shown in Figure 11.

The experimental results show that Schemes 1, 2, and 3 have missed detection for six kinds of defects, and the detection accuracy is low. Scheme 2 has overlapping detection frames for “inclusion” and “pitted_surface” defects, but the score of detection frames on these two defects is higher than that of Scheme 1. Scheme 3 has obvious overlapping detection frames for “patches” and “pitted_surface”, but the overall detection effect is better than Schemes 1 and 2. The defects of “inclusion,” “roll-in_scale,” and “scratches” are all detected in Scheme 4, but the “crashing” is still missed. The NMS algorithm was replaced by soft NMS on the basis of Scheme 4 in Scheme 5 to attenuate the nonmaximum detection frame score, rather than removing it completely by NMS. Therefore, the nonmaximum score detection frame is less suppressed compared with Schemes 1–3, and the detection of crazing defects is more accurate. The specific test results are shown in Table 5.

In Table 5, *a* is “crazing,” *b* is “inclusion,” *c* is “patches,” *d* is “pitted_surface,” *e* is “rolled-in_scale,” *f* is “scratches.” When the original Faster R-CNN model (Scheme 1) is used, the overall mean average accuracy is 62.4%. After replacing the original feature extraction network with ResNet-50, the average overall accuracy of Scheme 2 is 1.9% higher than that of Scheme 1, in which the average accuracy of “crashing,” “roll-in_scale,” and “scratches” is not improved significantly, but the accuracy of the pitted surface is improved to a certain extent. The mean average accuracy of Scheme 3 is 9% higher than that of the original Faster R-CNN, and 7.1% higher than that of Scheme 2, in which the average accuracy of “crashing” is the highest, which is 20.6%. The mean average accuracy of Scheme 4 is 12.1% higher than that of the original Faster R-CNN model, 3.1% higher than that of Scheme 3, and the average accuracy of “pitted_surface” is increased by 7.2%. The proposed algorithm (Scheme 5) is better than the original Faster R-CNN model and the average accuracy of “crashing” is reduced by 1.5% compared with Scheme 4, but the average accuracy of other defects is improved. The mean average accuracy is increased by 10.9%, 3.8%, and, 0.7%, respectively, compared with Schemes 2–4. We can see that the introduction of deformable convolution and deformable ROI pooling has a great impact on the network under the data in Table 5, which can improve the network's ability to extract complex features. It can be seen that the idea of fusing multiscale features with FPN has a certain positive impact on the network from the data of Scheme 4, and the effect is better than that of the model obviously with only deformable network. The scheme of replacing NMS with soft NMS reduces the average accuracy of crazing defects but improves the average accuracy on the whole. It also has a certain attenuation on the suppression of nonhighest detection frame and reduces the situation that defects with the large overlapping area are missed. Therefore, the combination of the deformable network and multiscale feature fusion scheme can improve the detection performance of the model.

### 2.9. Algorithm Comparison

The proposed algorithm is compared with other mainstream target detection algorithms in order to further verify the advantages of the proposed algorithm, as shown in Table 6. The comparison algorithms include single-stage target detection algorithms SSD and RetinaNet [38, 39] and double-stage target detection algorithm Cascade R-CNN [40].

In Table 6, *a* is “crazing,” *b* is “inclusion,” *c* is “patches,” *d* is “pitted_surface,” *e* is “rolled-in_scale,” *f* is “scratches.” It can be seen that the overall mean average accuracy of defects in SSD algorithm is only 51.0% from the data in Table 6, which cannot identify multiple defects effectively. The overall mean average accuracies of RetinaNet and Cascade R-CNN algorithms are all about 10% higher than that of SSD algorithm. The proposed algorithm has a great improvement in the detection accuracy of “crashing,” “inclusion,” “pitted_surface,” and “scratches” compared with other mainstream algorithms. The mean average accuracy of the proposed algorithm is 24.2%, 14.6%, and 12.6% higher than that of SSD, RetinaNet, and Cascade R-CNN, respectively. It shows that the proposed algorithm has a certain breakthrough in steel surface defect detection.

## 3. Conclusion

The surface defects of steel are taken as the research object in this paper. A new defect detection algorithm based on a deformable network combined with multiscale feature fusion algorithm is proposed in this paper in order to solve the problem of small size and complex shape of steel defect. (1) The deformable convolution is used to reconstruct the feature extraction network in order to enhance the network's ability to extract features. (2) Feature pyramid network is introduced to fuse the multiscale feature graph output to extract the deep semantic features of defect features. (3) The pooling of ROI is replaced by deformable pooling of ROI in order to improve the perception ability of the network to target defect location information. (4) The soft NMS method is used to alleviate the false detection and missing detection of the target. The proposed algorithm and other improved methods are tested on the NEU-DET dataset, and the detection effect is better than other improved methods. At the same time, the superiority of the proposed algorithm is verified by comparing with other mainstream target detection algorithms. The processed images in this paper could be affected by uncertainty. Therefore, it is necessary to use soft computing technology to improve image quality [41] in the future research. In addition, the detection time of this algorithm is long, so we need to further optimize the algorithm in the future to reduce the detection time. Based on the optimization of the algorithm, the fuzzy and similar defects will be deeply studied in order to improve the accuracy of defect detection further.

## Figures and Tables

**Table 1:** Hardware and software parameters of the experimental platform.

Name	Parameter
CPU	Intel core i7-6800 @ 3.4 GHz
GPU	NVIDIA GTX 1080Ti
Operating system	Linux Ubuntu 16.04
Deep learning framework	PyTorch 1.3.1
Language	Python 3.7.5

**Table 2:** Setting of the training parameter value.

Parameters	Learning rate	Weight decay	Momentum	Epochs	Pictures/GPU	Threads/GPU
Settings	0.02	0.0001	0.9	20	2	2

**Table 3:** The experimental data of the algorithm on all defects.

Defects	Crazing	Inclusion	Patches	Pitted_surface	Rolled-in_scale	Scratches
AP	0.501	0.791	0.792	0.874	0.649	0.905

mAP	**0.752**					

**Table 4:** Five schemes based on different improvement methods.

Schemes	ResNet-50	Deformable networks	FPN	Soft NMS
1	×	×	×	×
2	√	×	×	×
3	√	√	×	×
4	√	√	√	×
**5**	**√**	**√**	**√**	**√**

**Table 5:** The data results comparison of five schemes.

Schemes	AP						mAP	mAP increasing
	*a*	*b*	*c*	*d*	*e*	*f*		
1	0.250	0.652	0.752	0.735	0.545	0.811	0.624	−
2	0.252	0.677	0.772	0.789	0.555	0.816	0.643	+0.019
3	0.458	0.738	0.841	0.782	0.583	0.881	0.714	+0.090
4	0.516	0.754	0.768	0.854	0.610	0.902	0.745	+0.121
**5**	**0.501**	**0.791**	**0.792**	**0.874**	**0.649**	**0.905**	**0.752**	**+ 0.128**

**Table 6:** The algorithms of SSD, RetinaNet, and Cascade R-CNN are compared with the experimental results of the proposed algorithm.

Algorithms	AP						mAP	mAP increasing
	*a*	*b*	*c*	*d*	*e*	*f*		
SSD	0.302	0.522	0.620	0.390	0.709	0.515	0.510	+0.242
RetinaNet	0.391	0.676	0.772	0.711	0.546	0.542	0.606	+0146
Cascade R-CNN	0.321	0601	0.794	0.723	0.509	0.805	0.626	+0.126
This paper	**0.501**	**0.791**	**0.792**	**0.874**	**0.649**	**0.905**	**0.752**	−

## Data Availability

The program and image data used to support the findings of this study are available from the corresponding author upon request.
